# Production Assessment and Genome Comparison Revealed High Yield Potential and Novel Specific Alleles Associated with Fertility and Yield in Neo-Tetraploid Rice

**DOI:** 10.1186/s12284-020-00387-3

**Published:** 2020-06-03

**Authors:** Hang Yu, Muhammad Qasim Shahid, Qihang Li, Yudi Li, Cong Li, Zijun Lu, Jinwen Wu, Zemin Zhang, Xiangdong Liu

**Affiliations:** 1 State Key Laboratory for Conservation and Utilization of Subtropical Agro-Bioresources, South China Agricultural University, Guangzhou, 510642 China; 2 Guangdong Provincial Key Laboratory of Plant Molecular Breeding, South China Agricultural University, Guangzhou, 510642 China; 3 College of Agriculture, South China Agricultural University, Guangzhou, 510642 China; 4 Guangdong Laboratory for Lingnan Modern Agriculture, South China Agricultural University, Guangzhou, 510642 China

**Keywords:** *Oryza sativa*, Neo-tetraploid rice, Non-parental variation, Fertility, Yield

## Abstract

**Background:**

Neo-tetraploid rice (NTR) is a new tetraploid rice germplasm that developed from the crossing and directional selection of different autotetraploid rice lines, which showed high fertility and promising yield potential. However, systematic yield assessment, genome composition and functional variations associated with fertility and yield remain elusive.

**Results:**

Two season’s field trials of 15 NTRs and 27 autotetraploid rice (ATR) lines revealed that the improvement of YPP (yield per plant, 4.45 g increase) were significantly associated with the increase of SS (seed setting, 29.44% increase), and yield and seed setting of NTRs improved significantly compared to parental lines. Whole genome resequencing of 13 NTR sister lines and their parents at about 48.63 depth were conducted and genome compositions were illustrated using inherited chromosomal blocks. Interestingly, 222 non-parental genes were detected between NTRs and their low fertility parental lines, which were conserved in 13 NTRs. These genes were overlapped with yield and fertility QTLs, and RNA-Seq analysis revealed that 81 of them were enriched in reproductive tissues. CRISPR/Cas9 gene knockout was conducted for 9 non-parental genes to validate their function. Knockout mutants showed on an average 25.63% and 4.88 g decrease in SS and YPP, respectively. Notably, some mutants showed interesting phenotypes, e.g., *kin7l* (kinesin motor gene) and *kin14m* (kinesin motor gene), *bzr3* (BES1/BZR1 homolog) and *nrfg4* (neo-tetraploid rice fertility related gene) exhibited 44.65%, 24.30%, 24.42% and 28.33% decrease in SS and 8.81 g, 4.71 g, 5.90 g, 6.22 g reduction in YPP, respectively.

**Conclusion:**

Comparative genomics provides insights into genome composition of neo-tetraploid rice and the genes associated with fertility and yield will play important role to reveal molecular mechanisms for the improvement of tetraploid rice.

## Background

Rice is an important main food source for world’s population, and the yield of rice has long been the focus of breeders to feed growing population. The enrichment of genetic diversity can largely facilitate the improvement of rice cultivars (Huang et al. 2012; Wang et al. 2018). The phenomenon of whole-genome duplication is widespread throughout plant evolution (Soltis et al. 2007; Wood et al. 2009), and polyploid plants showed increased genetic variation that may provide a way to extend genetic diversity and breeding of elite cultivars (Wendel 2000; Soltis et al. 2009; Van de Peer et al. 2017).

Autotetraploid rice is a useful germplasm that generated by artificial genome duplication of diploid rice, which had biological advantages and wide adaptability (Shahid et al. 2011; Shahid et al. 2012; Wu et al. 2013; Tu et al. 2014; Yang et al. 2014). However, low fertility limited the yield performance of autotetraploid rice and its commercial utilization (Shahid et al. 2010; He et al. 2011; Shahid et al. 2013; Wu et al. 2014; Wu et al. 2015; Li et al. 2017; Chen et al. 2018b; Li et al. 2018; Ghouri et al. 2019). To solve this issue, rice scientists had made unremitting efforts for many years, and bred some new autotetraploid rice lines with high fertility (Tu et al. 2003; Luan et al. 2007; Cai et al. 2007; Guo et al. 2016; Guo et al. 2017). New male sterile lines, restorer lines and their F_1_ autotetraploid rice hybrids produced more than 70% seed setting (Tu et al. 2003; Luan et al. 2007). Cai et al. (2007) developed two polyploid rice lines with more than 65% seed setting. Two neo-tetraploid rice lines, Huaduo1 and Huaduo2 with high fertility (> 80% seed setting), were devolved from the crossing of autotetraploid rice by our research group in 2012 (Guo and Liu 2014). Other neo-tetraploid rice lines or hybrids, including Huaduo3, Huaduo4, Huaduo5, Huaduo8 and three F_1_ hybrids with high yield, including T449 × H1, T485 × H8 and H1 × H8, were also developed in recent years (Guo et al. 2017; Bei et al. 2019; Chen et al. 2019; Ghaleb et al. 2020). In contrast to autotetraploid and allotetraploid rice, neo-tetraploid rice (NTR) will play an important role in polyploid rice breeding because of high seed setting (> 70%), wide compatibility genes and stable fertility in next generations, like neo-tetraploid *Arabidopsis* (Yu et al. 2009; Guo and Liu 2014; Bei et al. 2019; Chen et al. 2019). Therefore, the systematic investigation of yield improvement, and the detection of specific DNA variations of neo-tetraploid rice will be helpful to decipher the high yield mechanism and optimize the directions of tetraploid rice breeding.

Next-generation sequencing is widely used for the detection of genomic variations and genome comparison analysis. Genome re-sequencing of three cultivars (sensitive cultivar IR64, drought resistance cultivar Nagina22, and salinity resistance cultivar Pokkali) with contrasting drought and salinity stress responses displayed 232 variant genes in known QTLs that may regulate abiotic stress (Jain et al. 2013). Genome re-sequencing of bulked populations facilitate rapid mapping of QTLs, and QTL-seq can be used to identify QTLs for important agronomic traits using F_2_ populations (Hiroki et al. 2013). Recently, the analysis of genome re-sequencing data of 3010 Asian cultivated rice accessions revealed about 30 million genomic variations, and the pan-genome was constructed, which provided a resource for rice genomics research and breeding (Wang et al. 2018). Deep genome re-sequencing provided high quality sequencing reads and reliable genomic variations, which allow the genome comparisons method to detect variation transmission patterns in pedigrees and identify the inherited blocks (Zhou et al. 2016). Whole genome sequencing of 30 cultivars revealed 28 chromosomal blocks inherited from ancestral lines that shared by all high-yielding cultivars (Huang et al. 2018). High resolution graphical genotype map was constructed for new upland African rice, which indicated similar genome constitution and common genetic events, and showed potential genes related to important agronomic traits by genome re-sequencing (Yamamoto et al. 2018).

In the present study, 18 morphological traits of 15 neo-tetraploid (NTRs) and 27 autotetraploid rice lines (ATRs) were observed during two seasons to illustrate the traits improvement between NTRs and their parental lines. Moreover, genome re-sequencing was employed to analyze the global DNA variations among 13 neo-tetraploid rice lines and their parental lines. Together with gene function and gene expression analysis, we planned to investigate the chromosomal composition map and to mine the functional variations that associated with fertility in neo-tetraploid rice. The fertility related genes in neo-tetraploid rice were knockout using CRISPR/Cas9 system, which validated the gene function and provided fundamental mutants for functional genomics research in tetraploid rice.

## Results

### Production Assessment of Neo-Tetraploid Rice (NTR)

**Fig. 1:**
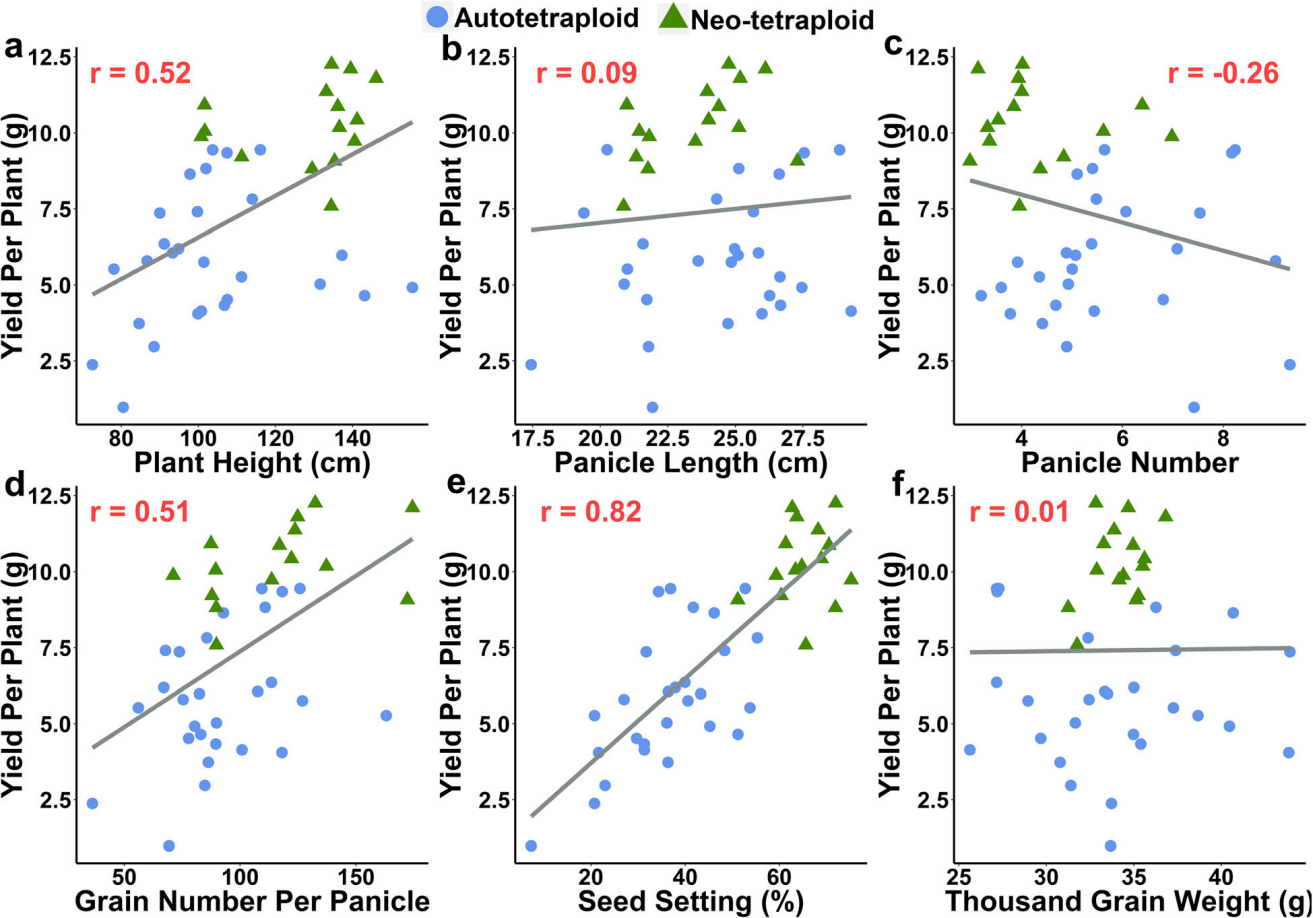
Distribution and correlation analysis of yield and yield related traits of 15 neo-tetraploid and 27 autotetraploid rice lines. Pearson correlation coefficient (*r*) is denoted by red font. The values of traits in this figure are the mean values of late season (2017) and early season (2018)

Firstly, we evaluated yield and yield related traits in 15 NTRs (Figure S1) and 27 autotetraploid rice (ATRs) lines, and the results showed that seed setting (i.e. average 65.32% in NTRs and 35.88% in ATRs), yield per plant (i.e. average 10.25 g in NTRs and 5.80 g in ATRs) and grain numbers per panicle (i.e. average 115.57 in NTRs and 93.31 in ATRs) improved significantly in NTRs (Table S1a and b). In order to confirm the factor that responsible for yield improvement in NTRs, correlation analysis between yield-related traits and yield per plant of 15 NTRs and 27 ATRs were conducted. The correlation coefficient was ranged from − 0.26 to 0.82, and seed setting (SS) showed the highest correlation coefficient (0.82), which indicated that SS contributed greatly to improve the yield in NTRs (Fig. 1).

Secondly, we compared NTRs with a famous diploid rice, Huanghuazhan (E285) and its autotetraploid rice, Huanghuazhan-4x (T485), for 18 agronomic traits during two seasons in the field. The results demonstrated that seed setting (SS) was the most improved trait in NTRs, and the average SS was significantly higher in NTRs than that in T485, and the highest SS (76.96%) was produced by Huaduo25100 during early season in 2018 (Figure S1 and Figure S2, Table S1a). The grain yield was significantly higher in NTRs than T485, and the average yield per plant (YPP) and yield per block (YPB) of NTRs were 10.25 g and 1.05 kg, while T485 produced 6.06 g and 0.59 kg, respectively. NTRs also showed improvement in grain number per panicle (115.57 in NTRs and 109.14 in T485), dry weight (32.52 g in NTRs and 29.83 in T485) and harvest index (0.32 in NTRs and 0.21 in T485). The yield of NTRs was not higher than diploid rice E285, however, yield of two hybrids, 4001-4x/Huaduo8 and Yangeng48-4x/Huaduo8, reached 26.52 g and 20.60 g, which was higher than E285 (20.44 g) (Table S1a, c).

Moreover, we also compared the field performance of NTRs under two different planting methods, viz. one seedling per hole (OSPH) and three seedlings per hole (TSPH). Under the planting method of TSPH, NTRs showed an earlier heading (2.89 days), higher plant height (2.67 cm), more number of panicles (0.44), higher dry weight (1.46 g), average yield per plant (0.5 g) and higher average yield per block (39.6 g) than OSPH (Figure S2).

**Fig. 2:**
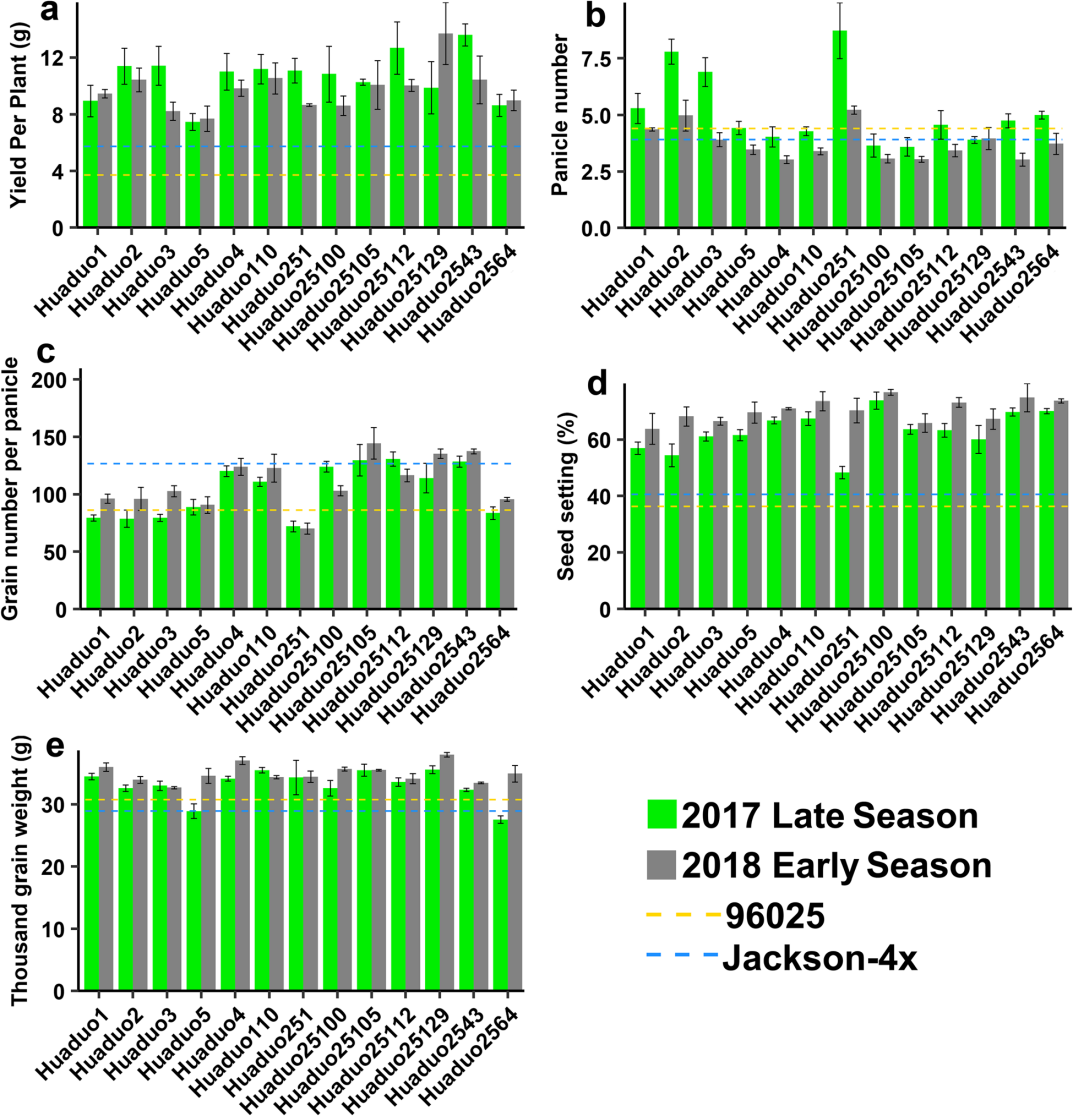
Comparison of yield and yield related traits between 13 neo-tetraploid rice (NTRs) lines and their parental autotetraploid rice (ATR) lines

Then, we compared the yield and yield components between 13 NTR sister lines and their parental lines (Figure S3), and NTRs displayed significant improvement in yield per plant, seed setting and thousand grain weight. The average grain yield per plant increased by 5.652 g in NTRs than their parental lines, which was mainly contributed by the improvement of seed setting (34.225% higher SS than their parental lines). Additionally, NTRs also increase in thousand grain weight by 4.726 g compared to their parental lines (Fig. 2). The significant improvement between NTRs and their parental lines prompted us to mine the genomic variations among them.

### Genome Re-Sequencing and Variation Detection in Neo-Tetraploid Rice

A total of 13 neo-tetraploid sister lines and their parental autotetraploid rice lines, Jackson-4x and 96025, were deeply sequenced by about 48.63 depth using genome re-sequencing, which generated about one billion high quality pair-end sequencing reads with an average ratio of Q30 score of 94.54% (Table S2), and the average genome coverage ratios for all samples were 95.48% of Nipponbare (MSU7) reference genome (Table S3). After removing of low-quality variations, with an average of about 1,849,640 variations were detected between these materials and reference genome (Table S4).

To test the reliability of these variations, two strategies were used to validate the variations. Firstly, the variations were validated by our previous genome sequencing data. Eleven individuals of tetraploid lines used in this study have been sequenced previously, the variation data were compared with the newly sequenced individuals, and the variation accordance rate was ranged from 83.14% to 94.39% with an average of 91.02% (Table S5**)**. Secondly, a total of 255 variations were validated using Sanger sequencing and 245 variations were in accordance with the re-sequencing data, and the accordance rate was 96.08% (Table S6). These results indicated that sequencing data and the detected variations were qualified and the variations are reliable.

### Genome Composition and Detection of Non-parental Chromosomal Blocks in Neo-Tetraploid Rice

**Fig. 3:**
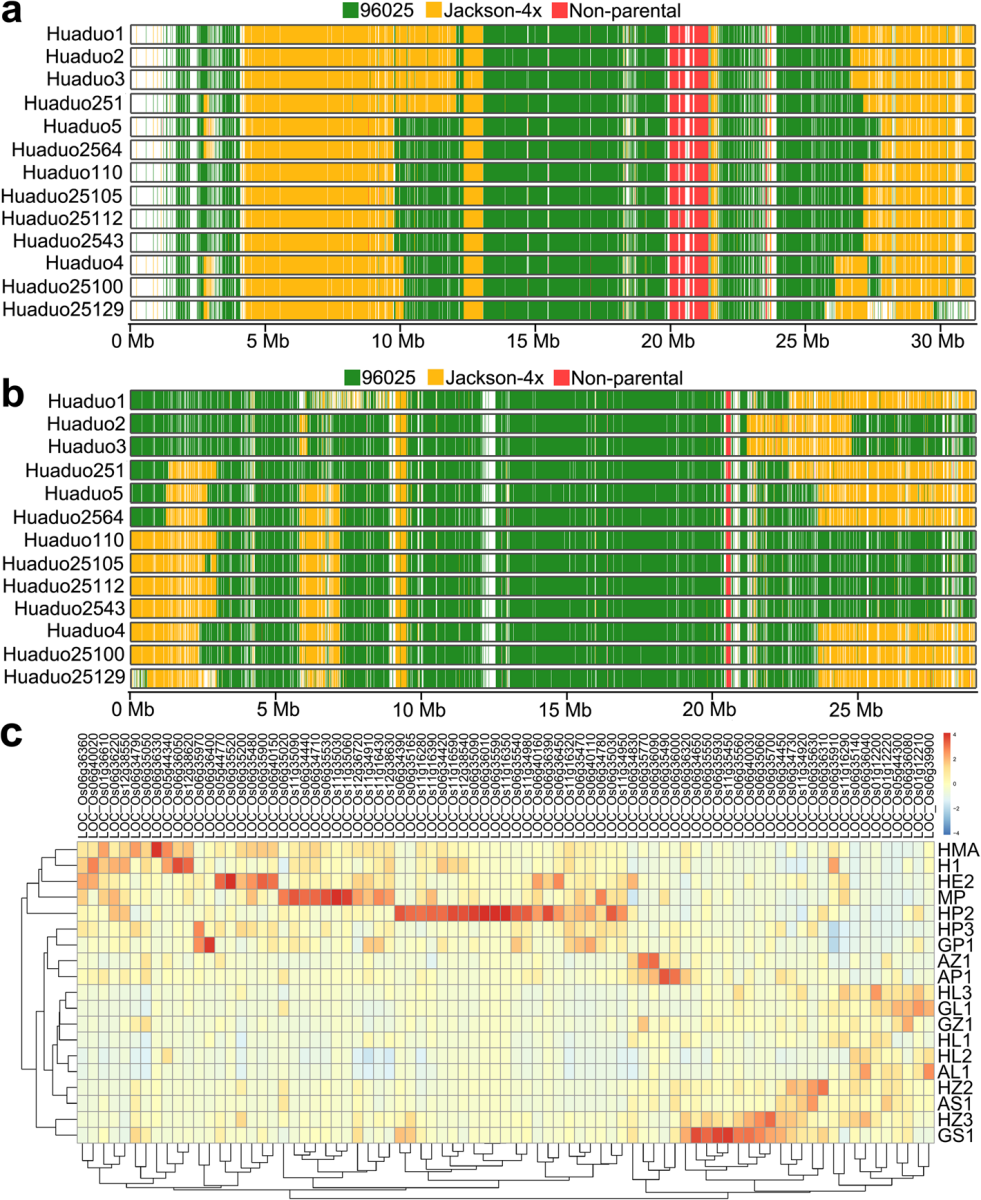
Inherited blocks inference of chromosome 6 (**a**) and chromosome 11 (**b**) of 13 NTR lines that developed from 96025 and Jackson-4x, and the expression pattern analysis of non-parental alleles in different tissues of neo-tetraploid rice, Huaduo1 (**c**) HMA, Anther and ovary in meiosis stage; H1, Anther in meiosis stage; HE2, ovary in flowering stage; MP, young panicle less than 5 mm; HP2, Anther in flowering stage; HP3, ovary at three days after flowering; GP1, sheath at five days after flowering; AZ1, branch and stem of panicle at flowering stage; AP1, sheath at flowering stage; HL3, flag leaf at three days after flowering; GL1, flag leaf at three days after flowering; GZ1, branch and stem of panicle at three days after flowering; HL1, flag leaf at meiosis stage; HL2 and AL1, flag leaf at flowering stage; HZ2, sheath at flowering stage; AS1, spikelet at flowering stage; HZ3, sheath at three days after flowering; GS1, spikelet at three days after flowering.

Genome composition analysis provided the information about inherited chromosome segments and the novel variations in neo-tetraploid rice lines. Graphical map showed the inherited genome composition of 13 NTR lines (Fig. 3, Figure S4). Most of the chromosomal blocks were inherited from either maternal (green) or paternal (gold) blocks in 12 chromosomes of NTRs. Interestingly, in chromosome 5, almost all chromosomal blocks were inherited form maternal line (96025) (Figure S4e), but in chromosome 10, almost all chromosomal blocks were inherited form paternal (Jackson-4x) (Figure S4i). We also observed non-parental chromosome blocks in 13 NTRs and the distribution of these blocks were similar among NTRs. A total of 140 non-parental chromosomal blocks that shared by all 13 NTRs were detected, which covered 1.4 Mb of rice genome (Table S7). Particularly, similar distribution was detected on chromosome 6 and 11, and almost same non-parental continuous blocks were identified in all 13 NTRs (Fig. 3a, b).

### Function and Expression Analysis of Non-parental Alleles in Neo-Tetraploid Rice

In these non-parental blocks, a total of 1080 nonsynonymous variations were detected in 222 genes. GO enrichment analysis revealed that these genes were enriched in FAD binding (Table S8). These variations were overlapped with the yield and fertility related QTLs. A total of 28 QTLs were overlapped with these variations, and 10 of them were yield related QTLs (such as biomass yield, spikelet number, panicle number and filled grain percentage) and 1 of them was sterility or fertility related QTL (Table S9). Results of QTL analysis indicated that non-parental variations maybe the key regions that controlled the yield and fertility in neo-tetraploid rice. Gene expression pattern analysis of these non-parental genes were conducted using two methods to further infer the function of these genes. Firstly, the expression levels of these genes were checked using RiceXPro database, and the expression information of 87 genes were recorded in this database, and 76 out of 87 genes were found to be expressed in the reproductive tissues including anther, pistil, ovary and embryo (Table S10). Secondly, using transcriptome data of neo-tetraploid rice line, Huaduo1, 81 genes were identified to be expressed in anther, ovary or flag leaf during meiosis or anthesis stage (Fig. 3c, Table S11). The genes that expressed in reproductive tissues may influence the reproductive process and seed setting rate of tetraploid rice. Interestingly, 64 genes were detected by both RiceXPro and our RNA-Seq data (Table S12).

### Function Validation and Knockout Phenotypes of Non-parental Alleles in Neo-Tetraploid Rice

**Fig. 4:**
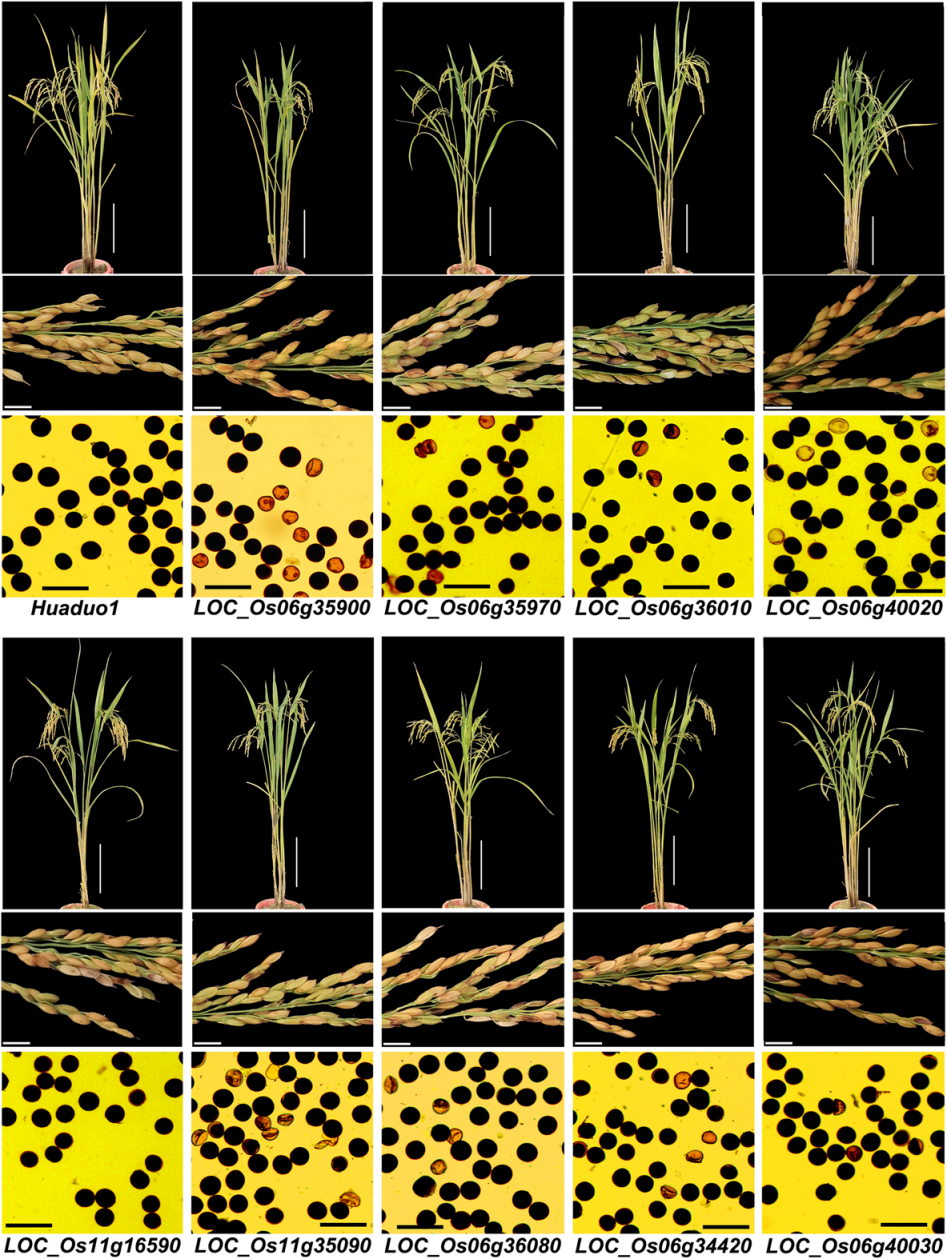
The non-parental allele knockout lines of neo-tetraploid rice showed different degrees of sterility. Knockout lines were developed by CRISPR-Cas9 system in neo-tetraploid rice line, Huaduo1. Scale bars: 20 cm for plant, 1 cm for panicle and 100 μm for pollen

In order to validate the gene function of the non-parental alleles, nine non-parental genes that expressed in reproductive tissues with unknown functions were selected for gene knockout by CRISPR-Cas9 system using a neo-tetraploid rice line, Huaduo1, with high pollen fertility (94.45%) and high seed setting (80.21%). These nine genes were *BZR3* (*LOC_Os06g35900*, https://www.uniprot.org/uniprot/?query=LOC_Os06g35900&sort=score), *CLPC3* (*LOC_Os11g16590*, https://www.uniprot.org/uniprot/?query=LOC_Os11g16590&sort=score), *KIN7L* (*LOC_Os11g35090*, https://www.uniprot.org/uniprot/?query=LOC_Os11g35090&sort=score), *KIN14M* (*LOC_Os06g36080*, https://www.uniprot.org/uniprot/B9FTR1), *LOC_Os06g35970* (it was named as *NRFG2* in the present study, i.e. *neo-tetraploid rice fertility related gene 2*), *LOC_Os06g36010* (it was named as *NRFG3* in the present study, i.e. *neo-tetraploid rice fertility related gene 3*), *LOC_Os06g40020* (it was named as *NRFG4* in the present study, i.e. *neo-tetraploid rice fertility related gene 4*), *LOC_Os06g34420* (it was named as *NRFG5* in the present study, i.e. *neo-tetraploid rice fertility related gene 5*) and *LOC_Os06g40030* (it was named as *NRFG6* in the present study, i.e. *neo-tetraploid rice fertility related gene 6*) (Table S13). The pollen fertility of knockout mutant lines in T1 generation was decreased by 9.52% to 56.96%, with an average of 26.71%. Knockout of one kinesin motor domain containing protein gene, *KIN7L* produced the lowest pollen fertility (37.49%) (Fig. 4, Table 1).

**Table 1:** Annotation of genes and phenotypes of CRISPR/Cas9 knockout mutants

Gene ID	Gene name	Gene Annotation	Pollen fertility (%)	Seed setting (%)	Yield per plant (g)
Huaduo 1	–	–	94.45 ± 1.67	80.21 ± 1.28	14.19 ± 0.78
*LOC_Os06g35900*	*BZR3*	BES1/BZR1 homolog protein, putative, expressed	64.33 ± 7.33^*^	55.79 ± 4.11^**^	8.29 ± 1.28^**^
*LOC_Os06g35970*	*NRFG2*	meiosis 5, putative, expressed	72.04 ± 12.75	60.87 ± 2.42^**^	10.77 ± 1.22^*^
*LOC_Os06g36010*	*NRFG3*	plastocyanin-like domain containing protein, putative, expressed	77.97 ± 5.95^*^	52.09 ± 4.43^**^	6.66 ± 0.92^**^
*LOC_Os06g40020*	*NRFG4*	DEAD-box ATP-dependent RNA helicase 52A, putative, expressed	84.93 ± 1.66^**^	51.88 ± 3.42^**^	7.97 ± 1.09^**^
*LOC_Os11g16590*	*CLPC3*	ATP-dependent Clp protease ATP-binding subunit clpA homolog CD4B, chloroplast precursor, putative, expressed	68.15 ± 11.31	57.43 ± 4.19^**^	11.74 ± 1.22
*LOC_Os11g35090*	*KIN7L*	kinesin motor domain containing protein, putative, expressed	37.49 ± 9.59^**^	35.56 ± 4.45^**^	5.38 ± 0.86^**^
*LOC_Os06g36080*	*KIN14M*	kinesin motor domain containing protein, expressed	77.81 ± 3.27^*^	55.91 ± 3.88^**^	9.48 ± 1.65^*^
*LOC_Os06g34420*	*NRFG5*	DEAD/DEAH box helicase domain protein, putative, expressed	54.05 ± 3.65^**^	67.13 ± 2.80^**^	11.99 ± 1.40
*LOC_Os06g40030*	*NRFG6*	S-locus-like receptor protein kinase, putative, expressed	72.92 ± 6.32^*^	54.58 ± 3.89^**^	11.59 ± 1.24

Huaduo1: a neo-tetraploid rice, wild type, control; *: *p*-value of t-test between 0.01 and 0.05; **: *p*-value of t-test less than 0.01

**Fig. 5:**
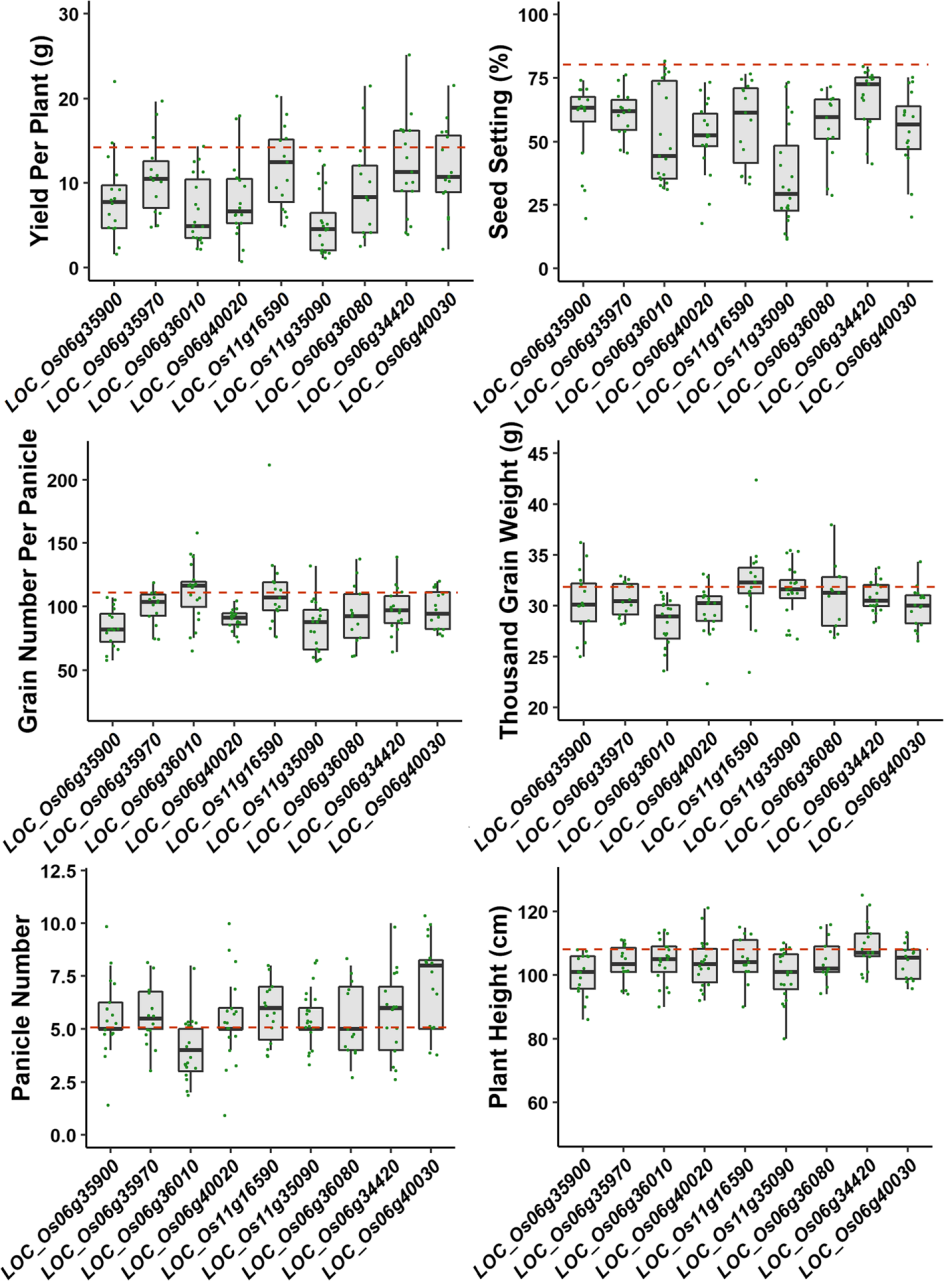
Evaluation of yield, yield components and plant height of nine CRISPR/Cas9 gene knockout lines in T1 generation. The dash line represents the value of trait of wild type Huaduo1

All knockouts showed an average of 25.63% reduction in seed setting, and the knockout of *KIN7L* showed the largest decrease in seed set (by 44.65%), while knockout of *NRFG5* showed the lowest decrease in seed set (by 13.08%). For yield per plant, nine knockouts showed an average of 4.87 g reduction in yield, and knockouts of four genes (*BZR3*, *NRFG3*, *NRFG4* and *KIN7L*) exhibited more than 5 g yield loss per plant. *kin7l* produced the highest lost in yield per plant by 8.81 g and *nrfg5* yielded the lowest lost in yield per plant by 2.20 g. In addition, other yield-related traits were also measured, 9 knockout lines showed 2.93 cm shorter in panicle length, 7 (except for *NRFG3* and *CLPC3*) knockout lines displayed 18.96 decrease in grain number per panicle, 8 (except for *CLPC3*) knockout lines showed 1.64 g reduction in thousand grain weight and 8 (except for *NRFG5*) knockout lines demonstrated 5.01 cm decrease in plant height compared with wild type. Interestingly, 8 (except for *NRFG3*) knockout lines showed an average of 0.72 increase in panicle number than wild type (Fig. 4, Fig. 5, Table 1, Table S15).

Of the 9 knockout genes, 3 genes exhibited important phenotype changes, such as knockouts of two kinesin motor domain containing gene, *kin7l* and *kin14m* decreased the seed setting by 44.65% and 24.30%, which cause yield loss of 8.81 g and 4.71 g per plant, respectively. Knockouts of BES1/BZR1 homolog *BZR3*, showed shorter plants (8.19 cm shorter than WT Huaduo1), shorter panicles (2.85 cm shorter than WT Huaduo1), less grains per panicle (27.82 less than WT Huaduo1) and lower seed setting than WT (24.42% less than WT Huaduo1) (Fig. 4, Fig. 5, Table S15).

## Discussion

### Improvement of Seed Setting Is the Main Contributor for Yield Increase in Neo-Tetraploid Rice

Low fertility of autotetraploid rice has long been the obstacle for tetraploid rice breeding. The investigation of 40 autotetraploid rice genotypes and their diploid counterpart revealed 46.24% reduction in seed setting, which cause significant loss in yield (Wu et al. 2013). Another study about 10 autotetraploid rice lines and their diploid counterpart also indicated significant loss in seed setting by 49.25% and 44.45% during early season and late season, which lead to 3.70 t/ha and 4.95 t/ha yield reduction (Shahid et al. 2013). Similar tendency was observed in other previous reports (Wu et al. 2014; Chen et al. 2018b). The development of neo-tetraploid rice has improved the seed setting up to 80% and overcome this limitation (Guo and Liu 2014; Guo et al. 2017). In this study, we further emphasized the significance of fertility improvement and thoroughly investigated the yield performance of neo-tetraploid rice. The correlation index between seed setting and yield per plant among 27 autotetraploid rice lines and 15 neo-tetraploid rice lines was 0.82, which indicating that seed setting is the main contributor for yield increase in neo-tetraploid rice. Other than fertility, correlation analysis also revealed that the grain number per panicle and plant height were also contributors for yield improvement, with correlation exponents of 0.51 and 0.52, respectively.

Furthermore, the systematic investigations of agronomic traits and field performance were conducted to evaluate the usage potential of neo-tetraploid rice and point out the direction of further tetraploid rice breeding. Guo et al. (2017) and Chen et al. (2019) evaluated 7 and 9 agronomic traits of neo-tetraploid rice lines and their hybrids. Another study investigated 7 agronomic traits of neo-tetraploid rice line Huaduo3, 66 and 134 (Bei et al. 2019). In this study, 15 neo-tetraploid rice lines were used and 18 agronomic traits were systematically assessed in field experiments of two seasons. All the lines were cultivated in randomized complete block design (RCBD) with three replications. Neo-tetraploid rice showed advantage in many traits compared to autotetraploid rice, such as grain yield, seed setting, grain number per panicle, dry weight and harvest index. The hybrids showed high potential for tetraploid rice breeding based on the high heterosis effect between NTRs and autotetraploid rice. Based on these results, we concluded that neo-tetraploid rice has the characteristics of high fertility, high yield and high hybrid vigor.

### Non-parental Alleles Play Important Role in the Fertility Improvement of Neo-Tetraploid Rice

In recent years, genome comparison based on high depth next generation sequencing data has been used to identify inherited chromosome blocks and key trait loci in several studies (Zhou et al. 2016; Huang et al. 2018; Yamamoto et al. 2018). The newly developed NTRs showed remarkable improvement in yield performance especially in fertility than their parental ATR lines. We speculated that the improvement in yield and seed setting might be due to the generation and selection of novel superior alleles that associated with high fertility and yield during breeding process for many years or the interaction between parental alleles. Our previous study detected new mutations in three neo-tetraploid rice lines (Bei et al. 2019). Here, we used comparative genomics between 13 neo-tetraploid rice sister lines and their parental lines to precisely illustrate the genome composition map of NTRs. The results of genome composition map provide useful information, such as most of the Chr5 regions were inherited from maternal line in NTRs, while most regions of Chr10 inherited from paternal line. This phenomenon of selective bias was previously reported in the HuangZaoSi-improved maize lines (Li et al. 2019) and Chr8 of “miracle rice” IR8 (Huang et al. 2018). Another promising result was the detection of 222 non-parental alleles and most of the non-parental genes were shared by 13 NTR sister lines, which indicated that these regions maybe the hotspot regions for genomic rearrangements as previously mentioned by Ramekar et al. (2015) in maize. Another potential reason for generation of novel mutations in NTRs is the enrichment or dosage effect of transposon elements (64 genes in non-parental regions were transposon element) in tetraploid rice genomes. These speculations may need further validation. The artificial selection of these novel variations in the following descendent generations showed that these variations were beneficial to the yield or fertility performance of NTR lines. These genes overlapped with yield and fertility related QTLs, and expressed in reproductive tissues, which indicated that they may regulate the fertility of polyploid rice.

Using CRISPR/Cas9 gene knockout system, the mutants of 9 non-parental genes were generated and they showed different extent of sterility, which validated the gene function. Kinesins are important intracellular transporter in microtubules, and *Pollen Semi-Sterility1* (*PSS1*) was previously reported to encode a kinesin-1-like protein that regulate male meiosis and fertility (Zhou et al. 2011). Two kinesin motor domain containing genes with non-parental and nonsynonymous variations in NTRs were detected, and their functions were confirmed to be associated with fertility by using CRISPR/Cas9 gene knockout. The seed setting of pss1 mutant was 40%, in this study, and the seed setting of 2 kinesin motor domain containing genes knockout lines, *LOC_Os11g35090* and *LOC_Os06g36080*, were 35.56% and 55.91%. Brassinosteroid (BR) genes, *D11* and *OsBZR1*, were identified to promote anther and seeds, and over-expression of these genes resulted in higher grain yield (Zhu et al. 2015). A BES1/BZR1 homolog protein gene, *LOC_Os06g35900*, was identified to be non-parental allele in NTRs and its knockout showed defected mature pollen and low yield. Moreover, knockout of other non-parental genes, such as two DEAD-box ATP-dependent RNA helicase genes, and a S-locus-like receptor protein kinase gene, also showed low seed setting. These results suggested that genome comparison among various materials is a valid method to detect candidate genes, and these mutants will be useful materials for further functional genome research of tetraploid rice.

## Methods

### Field Experimental Design and Investigation of Agronomic Traits

Fifteen neo-tetraploid rice lines (NTRs) were used in the present study (Table S1d), and all the NTRs showed stable morphological traits for several generations. Field experiments were performed at the experimental farm of South China Agricultural University during late season (LS, August to November) in 2017 and early season (ES, March to July) in 2018 under conventional field management. All 15 NTRs were planted under randomized complete block design (RCBD) with three replications. A famous diploid rice variety (Huanghuazhan) and its autotetraploid counterpart (Huanghuazhan-4x), which was developed from Huanghuazhan by chromosome doubling using colchicine treatment by our research group in 2016, were used as controls (CK). A total of 18 traits were investigated, including heading date (HD), plant height (PH), flag leaf length (FLL), flag leaf width (FLW), flag leaf length width ratio (FLLWR), panicle number (PN), panicle length (PL), grain number per panicle (GNPP), total grain number (TGN), seed setting (SS), thousand grain weight (TGW), grain length (GL), grain width (GW), grain length width ratio (GLWR), yield per plant (YPP), dry weight (DW), harvest index (HI), and yield per block (YPB). A total of 27 autotetraploid rice lines were planted at the same location during both seasons to compare with NTRs (Table S1d). Yield related traits, such as plant height (PH), number of panicles (PN), panicle length (PL), grain number per panicle (GNPP), total grain number (TGN), seed setting (SS), thousand grain weight (TGW) and yield per plant (YPP), were investigated.

### Analysis of Field Experiment Data

Multiple trait correlation analysis was conducted and visualized using the corrplot package (Wei and Simko 2017). To validate the potential heterosis effect of NTRs, one NTR line, Huaduo8, was used as paternal line to cross with 2 *indica*, 3 *japonica* and 1 *javanica* autotetraploid rice lines. The yield and yield components of the hybrid lines were investigated, and the mid parent heterosis (MPH) and over parent heterosis (OPH) were estimated based on the method described previously (Guo et al. 2017).

### Sample Preparation, Library Construction and Genome Sequencing

Young leaves of 13 NTRs and their parental lines (Jackson-4x and 96025) were collected and their genomic DNA were extracted using the cetyltrimethylammonium bromide (CTAB) method (Cota-Sanchez et al. 2006). The sequencing libraries were constructed, and sequenced based on the manufacturer’s instructions of Illumina HiSeq (Yu et al. 2018). The low-quality data were trimmed using the following criterions: (a) sequencing adapters were removed; (b) sequencing reads with N percentage more than 50% were removed; (c) sequencing reads with low-quality base (base quality value lower than 10) percentage more than 50% were removed. After filtration, the data quality was evaluated using the FastQC (v0.11.6) software (Andrews 2010).

### Reads Mapping and Variation Calling

The sequencing reads that passed the quality control process were mapped onto the MSU7 (Nipponbare, *O. sativa japonica*) reference genome using BWA (0.7.17-r1188) (Li and Durbin 2009), and MarkDuplicates in Picard (2.12.1) (Broad Institute 2019) were used to eliminate data of PCR duplication. The SAM files were sorted, indexed and converted to BAM format using SAMtools (version 1.9) (Li 2011). The genomeCoverageBed in bedtools (v2.27.1) was used to estimate the reference genome coverage (Quinlan and Hall 2010). Genome Analysis Toolkit (GATK, version 3.8–0) was used to call the variations from the alignment file, and the analysis pipeline was constructed based on the GATK best practices (Mckenna et al. 2010). The variations were annotated by SnpEff (4.3 s) based on the annotation GFF3 files of MSU7 reference genomes (Cingolani et al. 2012), and variations were compared by using VCFtools (0.1.16) software (Danecek et al. 2011). The software parameters for genome re-sequencing analysis are listed in Table S16.

### Genome Composition Analysis and Detection of Non-parental Alleles

A non-overlapped sliding windows method with step length of 10 kb was constructed using python script. Chromosome blocks were divided into three types: maternal, paternal and non-parental. Blocks with limited reliable markers (< 1 marker in one block) were excluded and there should be more than four markers in a block to determine non-parental block to increase the reliability. The blocks were retained if the block type was supported by at least 75% of markers in that block. Finally, the distributions of three types of blocks were plotted using ggplot2 (Wickham 2016). The non-parental alleles were detected using venn analysis provided by a toolkit of TBtools (Chen et al. 2018a). GO enrichment analysis was conducted using the agriGO v2.0 (Tian et al. 2017). During the update of Gramene database in 2010, the inferred location of rice QTLs were revealed by the sequences alignments of associated markers (Youens-Clark et al. 2011). QTL analysis was conducted by mapping the non-parental variations into the location of QTLs using our Python script that available at GitHub repository under the name of “extractQTLByPos”.

### Expression Pattern Analysis of Candidates

First, the expression levels of non-parental alleles were checked in the rice expression database, RiceXPro (Yutaka et al. 2010). Second, the expression information was analyzed using our transcriptome data. The transcriptome data were mapped onto the MSU7 (Nipponbare, *O. sativa japonica*) reference genome using STAR (2.7.1a) software (Alexander et al. 2013), and the expression matrix was generated using RSEM (v1.3.1) software (Li and Colin 2011). The expression data were illustrated using pheatmap (Raivo Kolde 2019).

### Validation of Variations Using Sanger Sequencing

Primers were designed using Primer Blast software in NCBI, and the segment of neo-tetraploid and autotetraploid containing variations were amplified and the PCR products were sequenced using Sanger sequencing. The sequences were aligned using ClustalW Multiple Alignment in BioEdit software.

### Construction of CRISPR/Cas9 Vector and Identification of Gene Knockout Mutants

Genome sequences of neo-tetraploid rice non-parental genes were used to design target-site primers using the targetDesign tool of CRISPR-GE (http://skl.scau.edu.cn/home/) (Table S13) (Xie et al. 2017). The target-site primers were located at the start of the exons to cause frameshift mutations. The CRISPR/Cas9 vectors were constructed as described previously (Butt et al. 2019; Ma et al. 2019), and CRISPR/Cas9 vectors were transferred into neo-tetraploid rice line, Huaduo1. The primers of gene-specific target site were used to select the transgenic plants, and the mutations were checked using Sanger sequencing. The sequencing data were decoded using DSDecodeM program of CRISPR-GE tool kit (Table S14) (Xie et al. 2017). The agronomic traits of about 15 plants for each gene mutant in T1 generation were measured, and the pollen fertility was observed by 1% I_2_-KI method using a microscope (Motic BA200).

## Conclusions

In this study, the agronomic traits of 15 neo-tetraploid rice lines were systematically investigated, and the improvement of yield and yield components, especially seed setting, were analyzed. Using comparative genomics between 13 neo-tetraploid sister lines and their parental lines, genome composition map of NTRs were illustrated and non-parental alleles were detected. The genes associated with reproductive tissues or development stages were detected and the gene functions were validated using CRISPR/Cas9 gene knockout. This study provided invaluable genomic resources, and the unique functional variations in NTRs will facilitate further marker assisted selection. The mutants of neo-tetraploid rice provide fundamental resources for functional genomics research in tetraploid rice.

## Supplementary information

**Additional file 1 Figure S1.** The performance of 15 neo-tetraploid rice (NTR) lines in field experiments. **Figure S2.** Yield performance and production assessment of neo-tetraploid rice lines compared with Huanghuazhan and Huanghuazhan-4x. **Figure S3.** Plant morphology of 13 NTRs and their parental lines. **Figure S4.** Inherited blocks inference in chromosome 1–5 (a-e), 7–10 (f-i), 12 (j) of 13 NTRs lines that developed from 96025 and Jackson-4x.

**Additional file 2 Table S1a.** Field performance of neo-tetraploid rice lines in 2017 late season (2017-LS) and 2018 early season (2018-ES). **Table S1b.** Average field performance of autotetraploid rice lines in 2017 late season and 2018 early season. **Table S1c.** Heterosis performance between neo-tetraploid and autotetraploid rice lines. **Table S1d.** List of the autotetraploid and neo-tetraploid rice materials used in this study. **Table S2.** Quality evaluation of genome sequencing data. **Table S3.** Mapping quality of sequencing reads to MSU7 reference genome. **Table S4.** Number of genomic variations against MSU7 reference genome. **Table S5.** Variation validation using previously sequenced individuals. **Table S6.** Validation of variations using Sanger sequencing. **Table S7.** Non-parental chromosome blocks that shared by 13 neo-tetraploid rice lines. **Table S8.** GO enrichment analysis of 222 non-parental genes in neo-tetraploid rice. **Table S9.** Reported QTLs that co-localized with non-parental variations in neo-tetraploid rice. **Table S10.** Non-parental genes that expressed in reproductive tissues using RiceXPro database. **Table S11.** Non-parental genes that expressed in reproductive tissues using RNA-Seq data of neo-tetraploid rice Huaduo1. **Table S12.** Non-parental genes that expressed in reproductive tissues in both RiceXPro and RNA-Seq data. **Table S13.** Gene annotation and primer sequences for CRISPR/Cas9 vector construction. **Table S14.** Genotype of CRISPR/Cas9 knockout lines of nine non-parental genes. **Table S15.** Pollen fertility and main agronomic traits of CRISPR/Cas9 knockout mutants. **Table S16.** Version, function and parameters of main software that used for genome re-sequencing and RNA-Seq data analysis.

## Data Availability

The raw reads of whole-genome resequencing were deposited at the NCBI Sequence Read Archive with accession ID PRJNA526117. The sequences and annotations of rice *japonica* reference genome MSU7 are available from the website http://rice.plantbiology.msu.edu/. All data supporting the conclusions described here are provided in tables, figures, and additional files.

## Abbreviations

NTR: Neo-tetraploid rice
ATR: Autotetraploid rice
SS: Seed setting
YPP: Yield per plant
RCBD: Randomized complete block design
QTL: Quantitative trait locus
OSPH: One seedling per hole
TSPH: Three seedlings per hole
GO: Gene ontology
*NRFG*: Neo-tetraploid rice fertility related gene
