# Supplementary material for: Production Assessment and Genome Comparison Revealed High Yield Potential and Novel Specific Alleles Associated with Fertility and Yield in Neo-Tetraploid Rice
Source: Rice (N Y). 2020 Jun 3;13:32. doi: 10.1186/s12284-020-00387-3 (PMC7271338; doi:10.1186/s12284-020-00387-3)
Supplement: Supplementary file 1 — Additional file 1 Figure S1. The performance of 15 neo-tetraploid rice (NTR) lines in field experiments. Figure S2. Yield performance and production assessment of neo-tetraploid rice lines compared with Huanghuazhan and Huanghuazhan-4x. Figure S3. Plant morphology of 13 NTRs and their parental lines. Figure S4. Inherited blocks inference in chromosome 1–5 (a-e), 7–10 (f-i), 12 (j) of 13 NTRs lines that developed from 96025 and Jackson-4x. [file 12284_2020_387_MOESM1_ESM.pdf]

Additional file 1

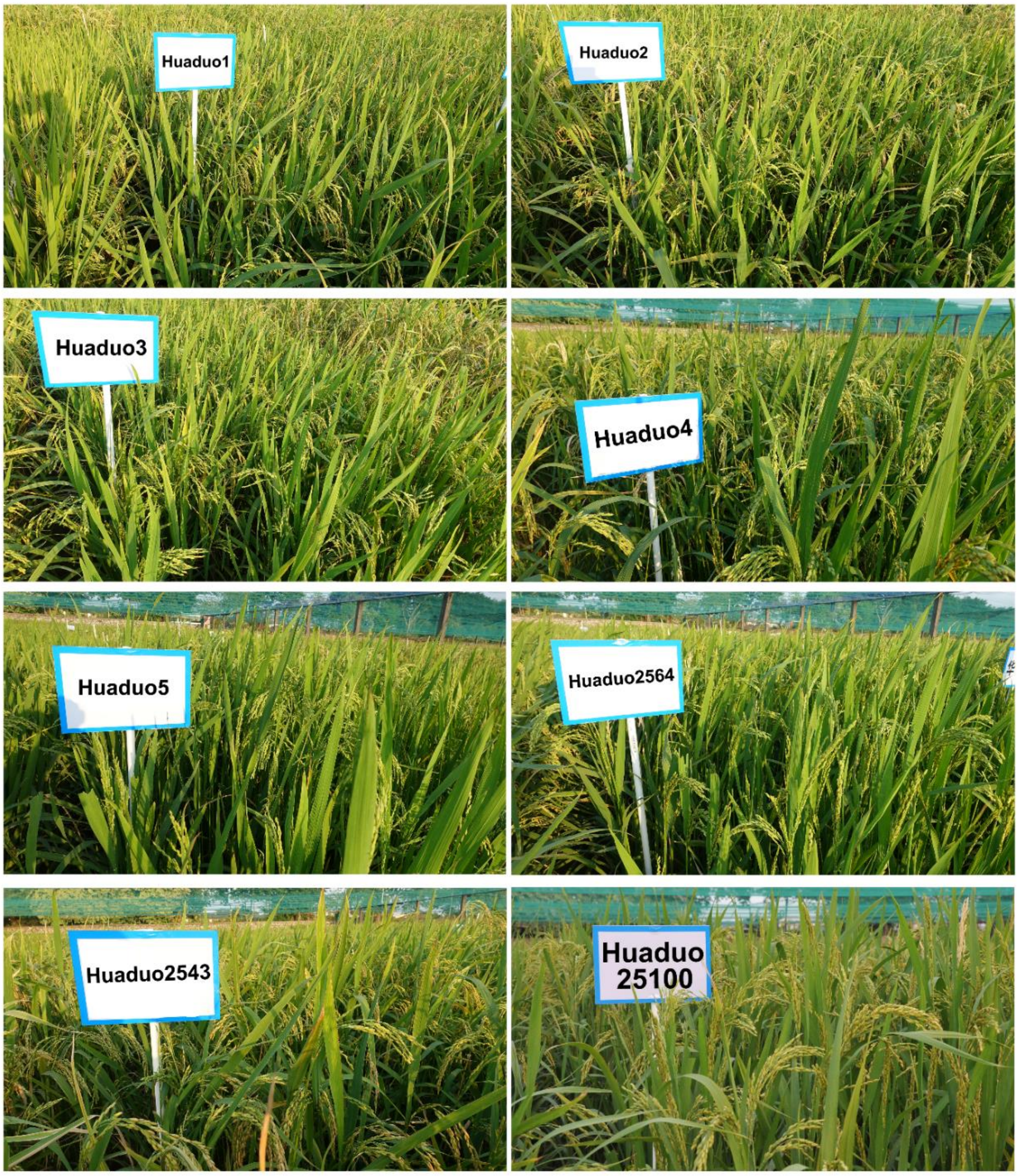

Figure S1a The performance of 15 neo-tetraploid rice (NTR) lines in field experiments.

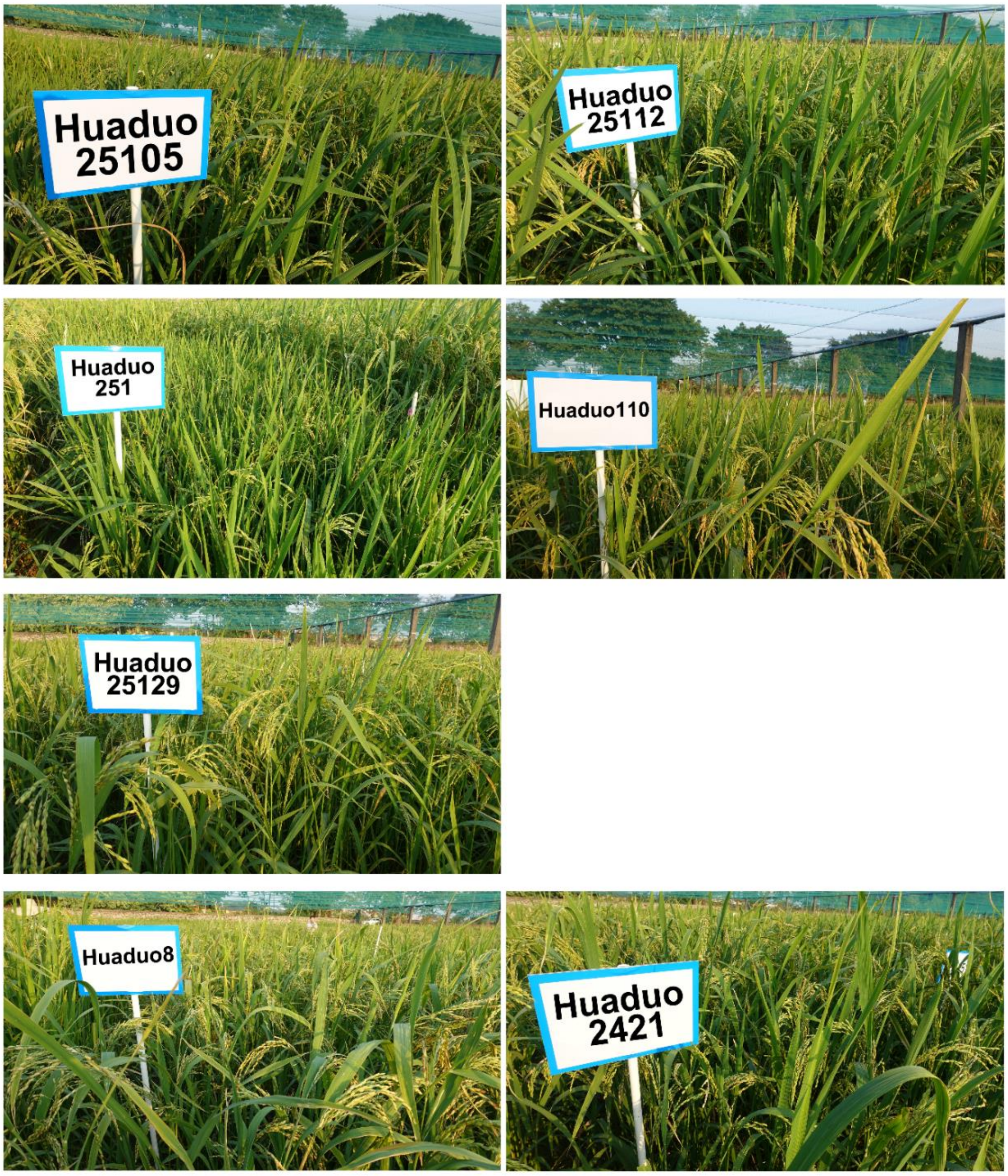

Figure S1b The performance of 15 neo-tetraploid rice (NTR) lines in field experiments.

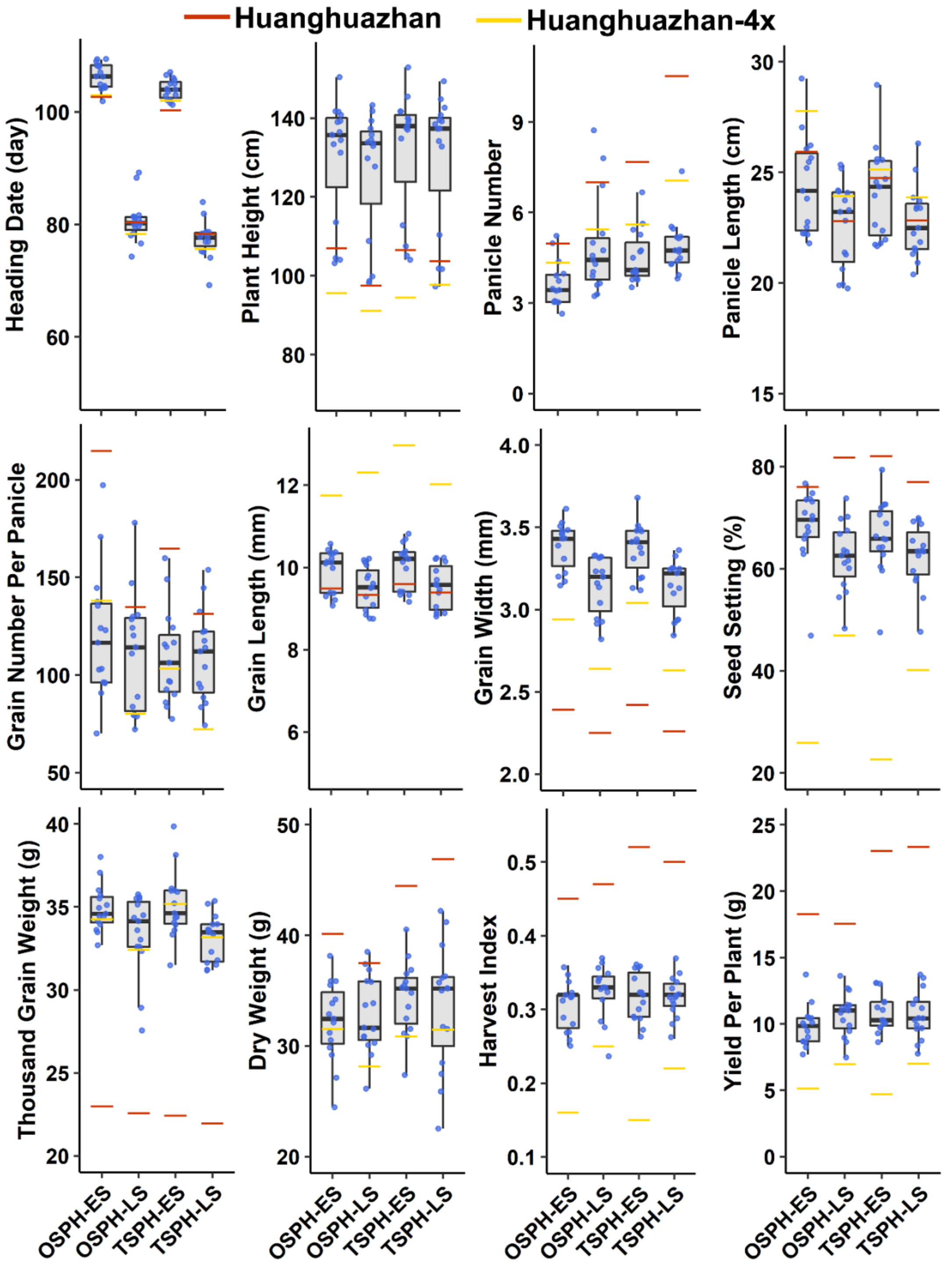

Figure S2 Yield performance and production assessment of neo-tetraploid rice lines compared with Huanghuazhan and Huanghuazhan-4x.

The average values of traits with three replications were used. OSPH, one seedling per hole; TSPH, three seedlings per hole; ES, early season; LS, late season. The blue dots indicate the values of traits of neo-tetraploid rice lines.

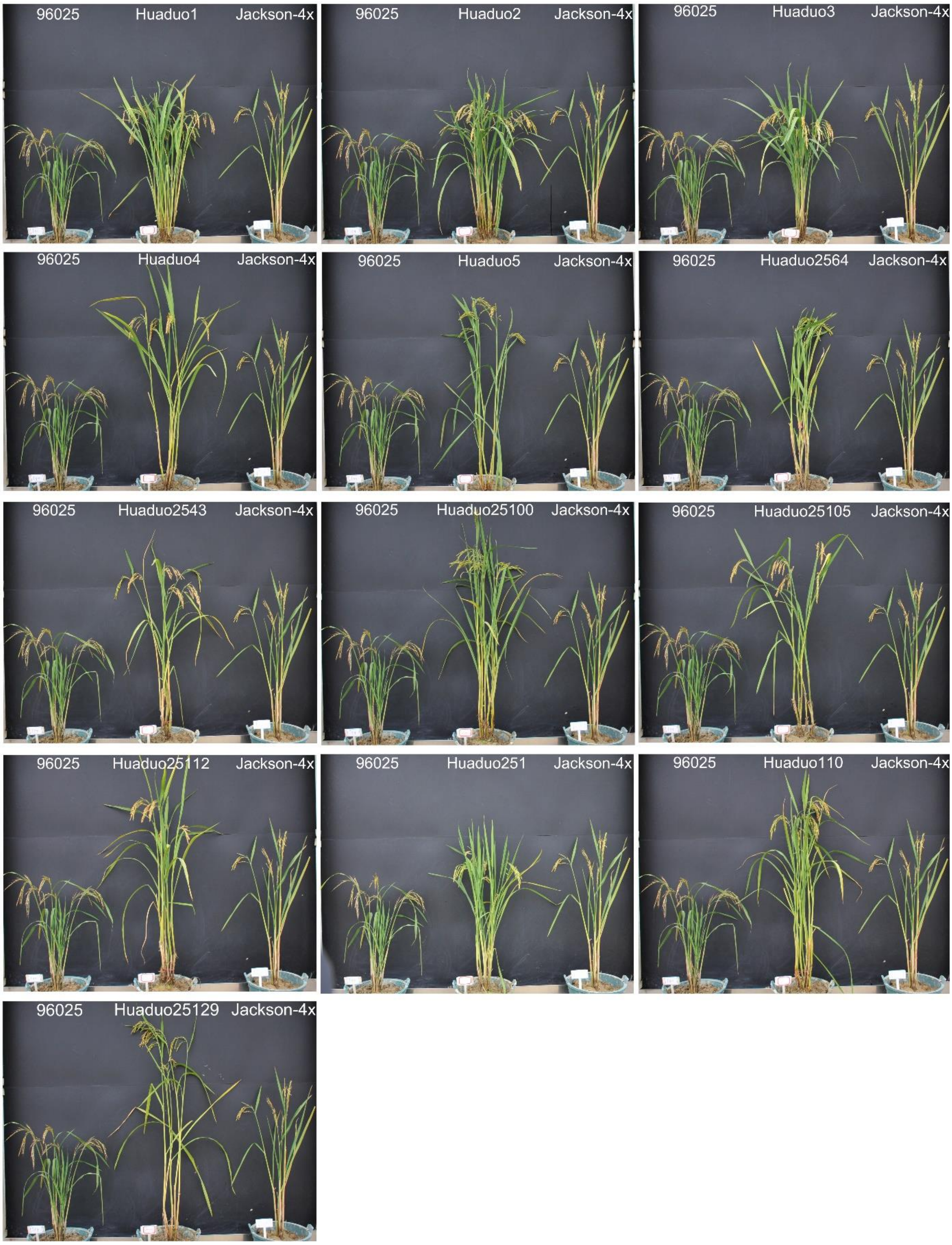

Figure S3 Plant morphology of 13 NTRs and their parental lines.

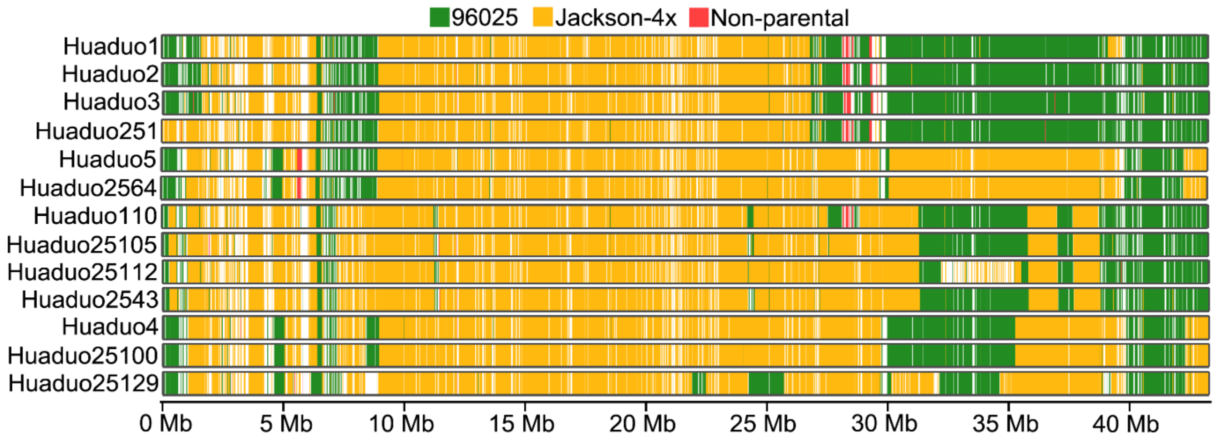

Figure S4a Inherited blocks inference in chromosome 1 of 13 NTRs lines that developed from 96025 and Jackson-4x.

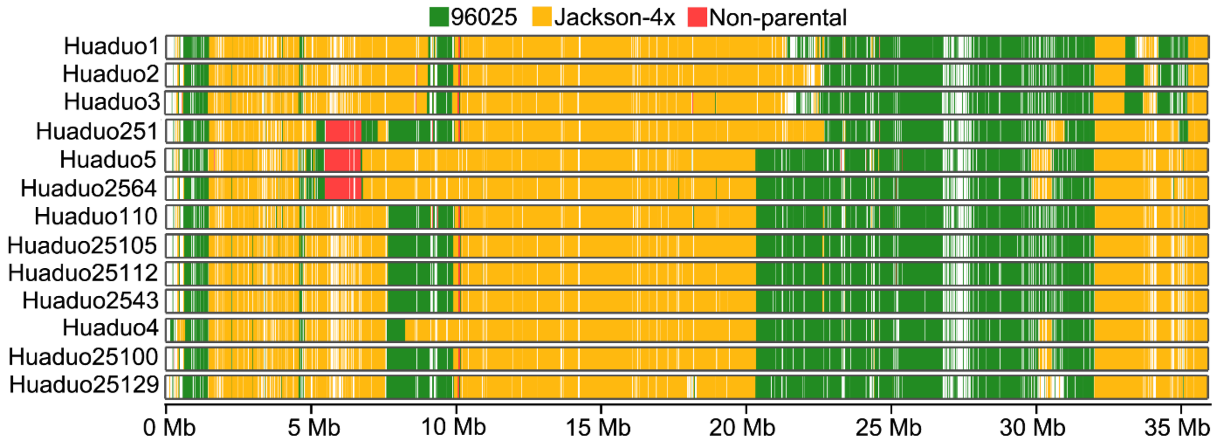

Figure S4b Inherited blocks inference in chromosome 2 of 13 NTRs lines that developed from 96025 and Jackson-4x.

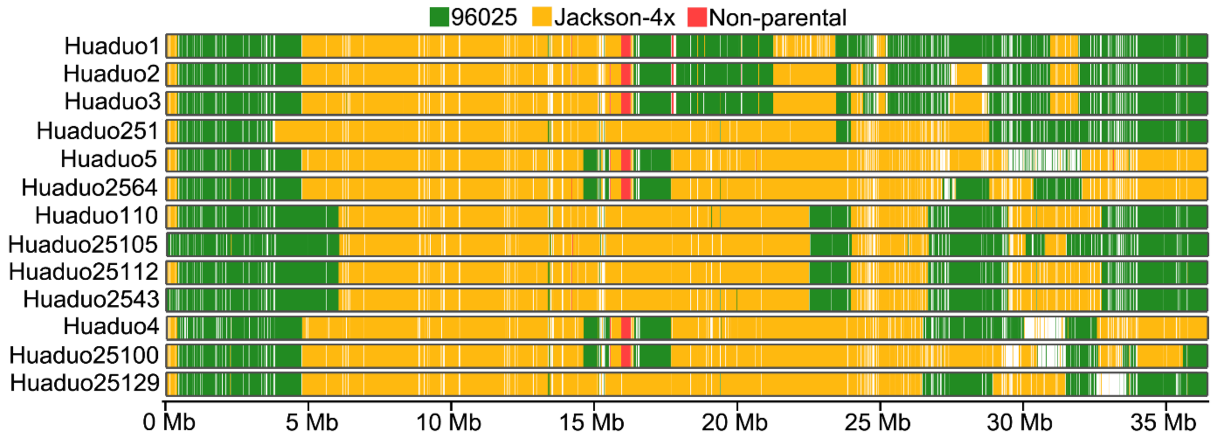

Figure S4c Inherited blocks inference in chromosome 3 of 13 NTRs lines that developed from 96025 and Jackson-4x.

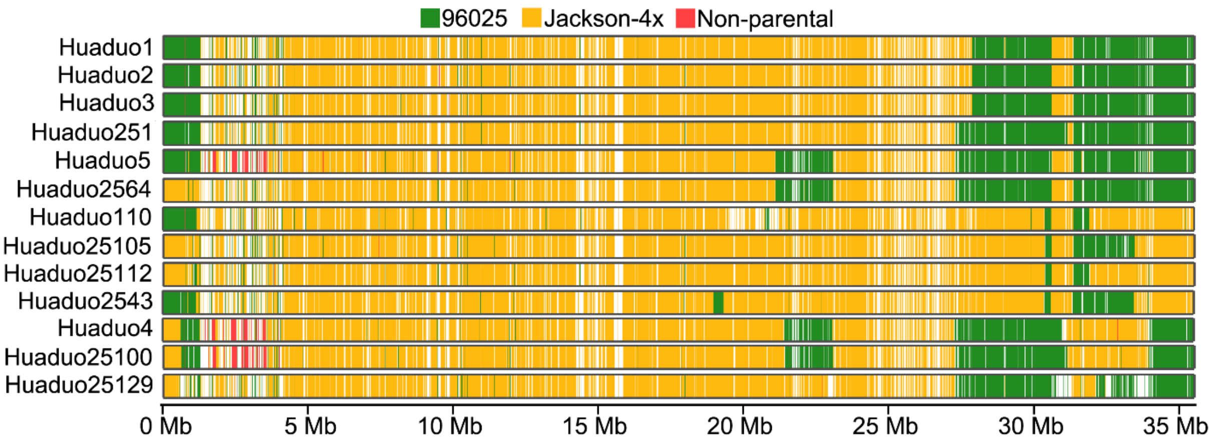

Figure S4d Inherited blocks inference in chromosome 4 of 13 NTRs lines that developed from 96025 and Jackson-4x.

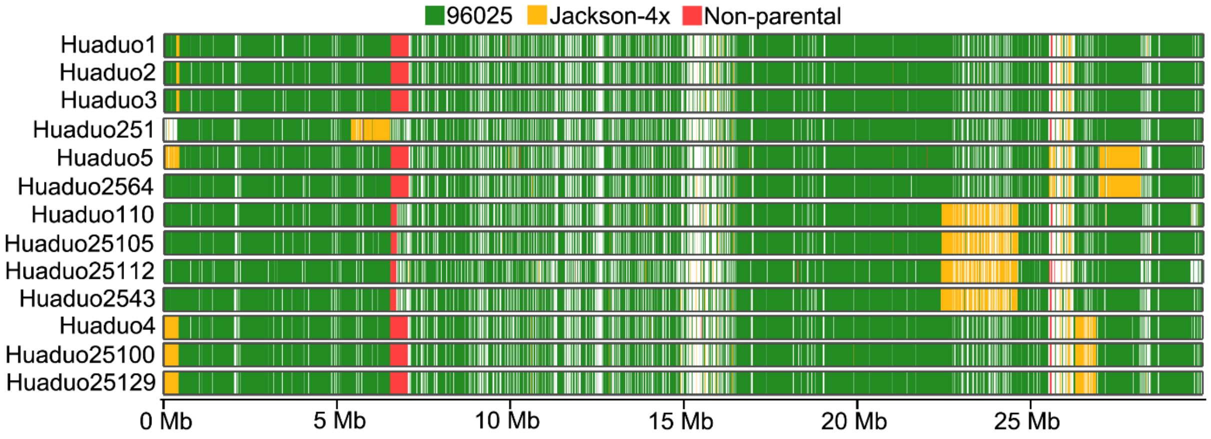

Figure S4e Inherited blocks inference in chromosome 5 of 13 NTRs lines that developed from 96025 and Jackson-4x.

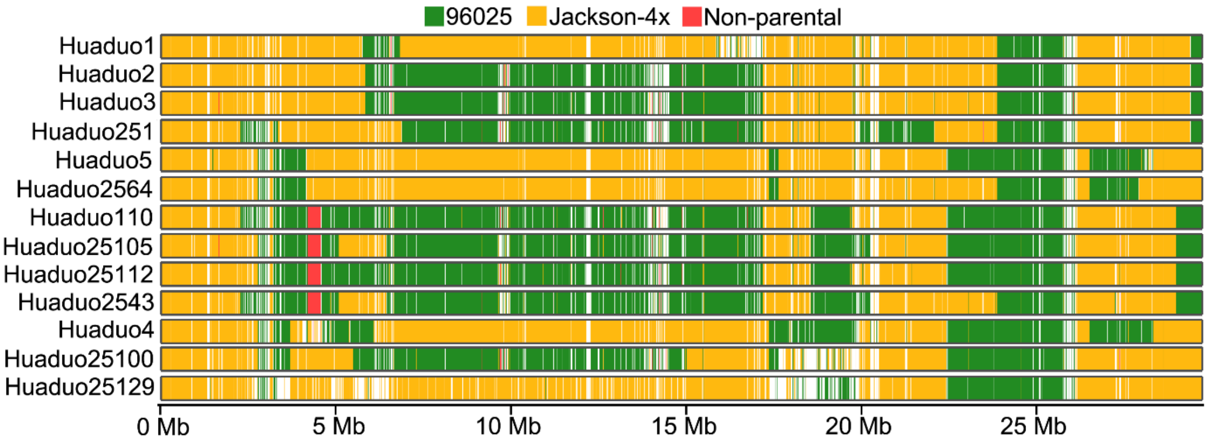

Figure S4f Inherited blocks inference in chromosome 7 of 13 NTRs lines that developed from 96025 and Jackson-4x.

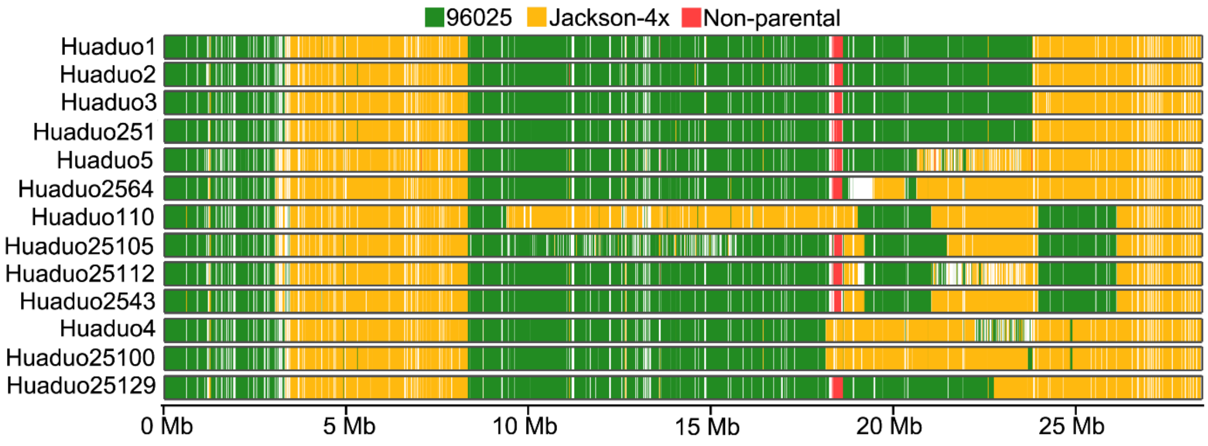

Figure S4g Inherited blocks inference in chromosome 8 of 13 NTRs lines that developed from 96025 and Jackson-4x.

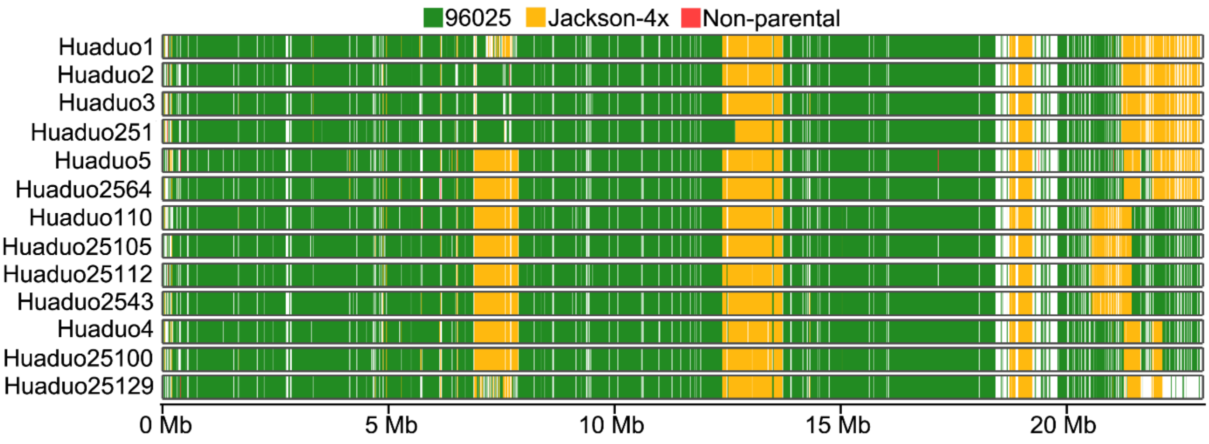

Figure S4h Inherited blocks inference in chromosome 9 of 13 NTRs lines that developed from 96025 and Jackson-4x.

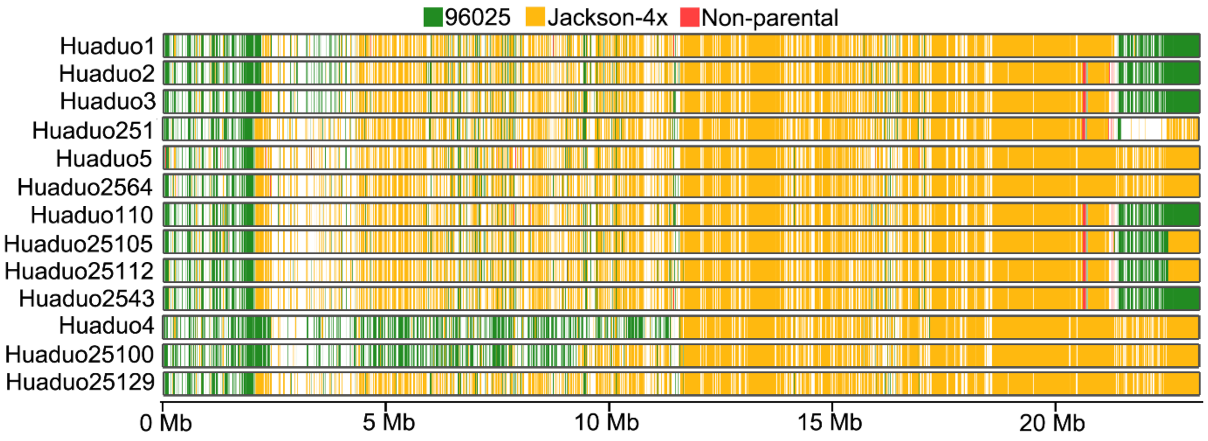

Figure S4i Inherited blocks inference in chromosome 10 of 13 NTRs lines that developed from 96025 and Jackson-4x.

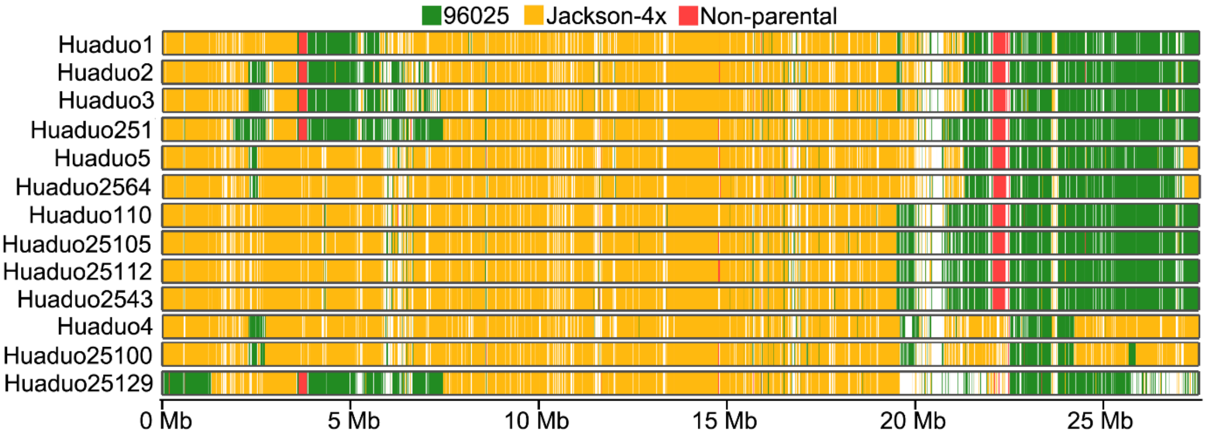

Figure S4j Inherited blocks inference in chromosome 12 of 13 NTRs lines that developed from 96025 and Jackson-4x.
